# Changing incidence of bovine babesiosis in Ireland

**DOI:** 10.1186/2046-0481-67-19

**Published:** 2014-09-05

**Authors:** Annetta Zintl, Guy McGrath, Luke O’Grady, June Fanning, Kevin Downing, Denise Roche, Mícheál Casey, Jeremy S Gray

**Affiliations:** 1 UCD School of Veterinary Medicine, University College Dublin, Dublin, Ireland; 2 Central Veterinary Laboratory, Department Agriculture, Food & the Marine, Backweston, Celbridge, Co Kildare, Ireland; 3 Irish Cattle Breeding Federation, Bandon, Co Cork, Ireland; 4 Growth from Knowledge, GfK Kynetec Ltd, 2 Weston court, Weston, Newbury, Berkshire RG20 8JE, UK; 5 School of Biology and Environmental Science (Emeritus Professor), University College Dublin, Dublin, Ireland

**Keywords:** Tick-borne parasite, Redwater fever, Bovine babesiosis, Farm survey, Babesia divergens

## Abstract

**Background:**

In Ireland bovine babesiosis is caused by the tick-borne blood parasite, *Babesia divergens*. A survey of veterinary practitioners and farmers in the 1980’s revealed an annual incidence of 1.7% associated with considerable economic losses. However, two subsequent surveys in the 1990’s indicated a decline in clinical babesiosis. Recent evidence from continental Europe suggests that, probably due to climate change, the distribution of the tick vector of *B. divergens*, *Ixodes ricinus* is extending to more northerly regions and higher altitudes. In addition, milder winters are thought to widen the window of tick activity.

In order to determine whether any such changes have affected the incidence of bovine babesiosis in Ireland, a questionnaire survey of farmers and veterinarians was carried out and compared with data from previous surveys.

**Results:**

Our survey indicates that while the incidence of clinical disease has continued to decline, cases can occur at any time of year. In contrast to previous surveys, affected farms were the same size as unaffected ones. There was no correlation between disease risk and the presence of deer on the land. Disease severity and mortality rates were increased because many infections were advanced by the time they were detected and treated.

**Conclusion:**

While the precise reasons for the decline in the incidence of redwater are unknown, changes in agricultural practice are likely to be of importance. A reversal of the trend could be devastating, as vigilance among farmers and veterinarians is flagging and the national herd is losing its protective immunity to disease.

## Background

*Babesia divergens* is a protozoan parasite that causes redwater fever in cattle. In the early stages of infection, the intra-erythrocytic parasite causes progressive haemolytic anaemia, resulting in haemoglobinaemia and haemoglobinuria, to which the disease owes its name. Affected animals present with high fever, listlessness, dehydration and ‘pipestem’ diarrhea [1,2]. In the absence of treatment the infection progresses to severe haemolytic anaemia. Animals become constipated and body temperatures fall to subnormal levels. During the terminal stage of disease animals exhibit hypoxia/anaemia and toxaemic shock. The pulse becomes weak and jaundice can be severe. While most animals are recumbent at this stage, some may show excitability or aggressive behavior [3].

The only antibabesial product on the market in most of Europe today is imidocarb diproprionate (Imizol, MSD Animal Health). To be fully effective, treatment must be initiated during the early stage of infection. Side effects of the product include coughing, muscular tremors, salivation, colic and irritation at the injection site [4]. Once the infection has advanced to acute anaemic anoxia, the prognosis is poor and blood transfusion is recommended [2]. Imidocarb is also an effective prophylactic at twice the therapeutic dose. However, long withdrawal periods (213 days for meat and 21 days for milk) must be observed [5].

*B. divergens* is transmitted by the hard tick, *Ixodes ricinus. I. ricinus* is a three-host tick and its life cycle generally takes about three years to complete, with each instar engorging just once before moulting to the next life cycle stage. While the tick tolerates a broad temperature range (the threshold temperature for both development and activity is 7 to 10°C) it is highly susceptible to desiccation, and requires humidity levels of at least 80% [6]. As a result *I. ricinus* is mainly found in rough hill scrub, unimproved under-grazed pastures and damp low-lying land that does not dry out during the summer or flood during the winter. Pockets of populations may also survive in hedges and headlands [7]. In endemic areas where tick densities are high, the incidence of clinical disease is generally low. This so-called ‘enzootic stability’ is due to the phenomenon of inverse age resistance which renders calves up to 9 to 12 months of age relatively resistant to disease [8]. These animals develop fully protective immunity before their innate resistance wears off. In contrast, in areas where tick populations are patchy, a proportion of cattle grow up without becoming exposed. These are fully susceptible and likely to suffer severe redwater fever when infected.

Several decades ago, bovine babesiosis caused by *B. divergens* was considered one of the most important tick-transmitted diseases in cattle [9]. In Ireland the parasite was considered of such clinical significance that a live attenuated vaccine was developed and used experimentally to successfully immunise approx. 14,000 cattle in local trials ( [10], J.S. Gray personal communication). Since then there has been growing evidence that the prevalence of the disease, if not that of the parasite is declining [11-13]. These observations are surprising particularly in light of a growing body of research that suggests that, due to climate change, the tick vector *I. ricinus* is extending its range [14-16].

This article reports on the current status of redwater fever in Ireland and how the epidemiology of the disease may be changing.

## Methods

### Survey design and dissemination

Using the software programme SurveyMonkey ( http://www.surveymonkey.com) two questionnaires (one targeted at farmers and one at veterinary practitioners) were designed to obtain information on the number and presentation of clinical babesiosis cases in the preceding 12 months. Further questions referred to perceived changes in the incidence or severity of bovine babesiosis over the last decade as well as the methods commonly used to identify infections. In addition effectiveness of treatment and prophylaxis were assessed. Finally farmers were asked to provide general information on their herd (such as size and type) and their farm (implementation of environmental protection measures and the presence of deer) as well as the first 4 digits of their herd number. These data provided information on the approximate location of the farm without compromising confidentiality. While the questionnaire that was distributed to veterinarians could not deliver actual figures on prevalence, it was used to identify infection ‘hotspots’ in the country. Both questionnaires are provided in the appendix of the online version (Additional files 1 and 2).

Short advertisements were sent to national farming and veterinary journals and organisations to promote participation in the survey (including the Irish Farmers Journal, Irish Cattle Breeders Federation, Irish Veterinary Journal, Veterinary Ireland and the UCD School of Veterinary Medicine mailing list). In addition, surveys were distributed at three national veterinary conferences. Responses were received between April and September 2013.

### Statistical analysis

Using the information provided in the farm survey, herds were categorized into ‘dairy’ (those with 70% or more of all adult animals listed as dairy cows), ‘beef’ (those with 70% or more listed as beef cattle) and ‘mixed’ (where neither beef nor dairy animals predominated). Moreover farms were grouped into four herd sizes with the smallest herd ranging from 1 to 99 animals, followed by 100 to 199 and 200 to 299 cattle. The largest herds comprised 300 head or more. Farms that had experienced cases of bovine babesiosis in the previous 12 months were compared against those that had not using Chi-square analysis. Numbers of animals bought in over this period were compared by Mann Whitney U test. Differences were considered significant at p < 0.05.

## Results

### Overall incidence of clinical babesiosis

**Figure 1:**
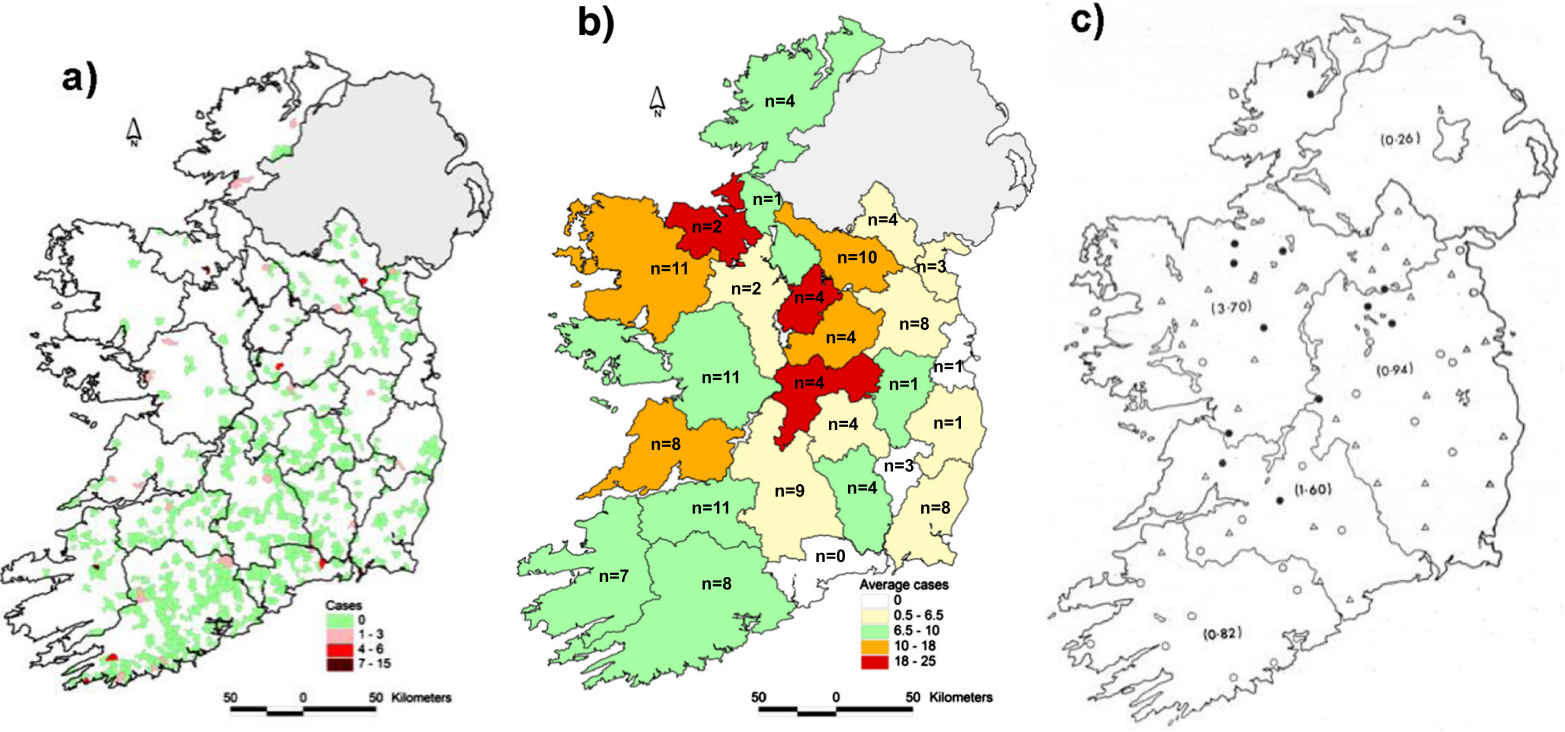
**Geographical distribution of clinical babesiosis cases as reported by (a) farmers and (b) veterinary practitioners in the present survey and (c) in surveys carried out by Gray and Harte **[19]** (reproduced with kind permission of the Irish Veterinary Journal).** 1**a**: The unit of representation is a District Electoral Division, colours indicate the number of cases that occurred on each farm in the last 12 months; 1**b**: Colours indicate the average number of cases attended by each veterinarian in the last 12 months, n indicates the number of responses per county; 1**c**: Figures in brackets represent percentage incidence according to the farm survey and Taylor [26], circles and triangles represent clinical babesiosis cases treated by veterinary practices (open circles: <100 cases per year, triangles: 100–300 cases per year; closed circles: >300 cases per year).

A total of 721 farm responses were received. Using criteria described in the Methods, the majority of responding farms were categorized as ‘dairy’ (83%, n = 599) and 16.5% as ‘mixed’ (n = 119). Only three farms qualified as ‘beef farms’. The total number of cattle on all surveyed farms was estimated to be about 156,578.Overall 33 respondents (4.6%) had experienced cases of clinical babesiosis on their farms in the previous 12 months. With a total of 91 redwater cases, the estimated incidence rate was 0.06%. Cases were reported country-wide (Figure 1a) with no obvious foci of infection. The only possible exception to this was an area on the southwest coast where several farms reported incidences of 4 to 6%. Most responses were received from the south and the east of the country, where cattle farmers, particularly those involved in dairy, are concentrated. Coverage of the west and the north where beef and sheep farming predominate, was scantier.In total 142 veterinary practitioners participated in the survey. Responses were received from all counties in Ireland but one (Waterford) (Figure 1b). The numbers of cases attended by each veterinarian ranged widely across the country (0 to 50) with most cases occurring in counties on the west coast and east of the River Shannon. The average case rate overall was approximately 10 per veterinary practitioner.

Twenty-three percent of farmers and 62% of veterinarians found that the number of babesiosis cases had decreased in the last 10 years. Only a very small number of respondents (2.6% of farmers and 6.5% of veterinarians) reported an increase in cases over the same period of time. The remainder either felt that there was no change (33% of farmers, 14% of veterinarians) or they did not know (41% of farmers and 17% of veterinarians).

### Characteristics of farms with and without babesiosis cases

There was no difference in size between farms that had experienced babesiosis cases in the last 12 months and those that had not (Table 1). Over this time period, farms had bought in 5.3 animals on average. This number was considerably, though not significantly, lower in farms with cases than in farms without cases.

**Table 1:** Comparison of farms with and without bovine babesiosis

**Farm characteristics (number of responses)**	**(n = 721) Farms with clinical cases**	**Farms without clinical cases**	**Statistical significance**
**Herd size**			
1-99 cattle	8 (24.2%)	91(13.2%)	n.s. (χ2 = 6.19; df = 3; p = 0.1)
100-199 cattle	11 (33.3%)	312 (45.3%)	
200-299 cattle	4 (12.1%)	141 (20.5%)	
≥ 300 cattle	10 (30.3%)	144 (21.0%)	
Number of bought-in animals over the last 12 months	1.75 animals/farm	5.5 animals/farm	n.s. (W = 242521.5; p = 0.08)
Former Rural Environmental Protection Scheme (REPS) participant (n = 713)	25 (75.8%)	366 (53.8%)	p < 0.05 (χ2 = 6.11; df = 1)
Presence of a habitat under special environmental protection (SAC, SPA, NHA) (n = 625)	6 (22%)	74 (12.4%)	n.s. (χ2 = 2.2; df = 1; p = 0.1)
**Presence of deer on the farm (n = 714)**			
Deer observed			
- Very often to occasionally (weekly, monthly, every couple of months)	4 (12.1%)	96 (14.1%)	n.s. (χ2 = 0.16; df = 2; p > 0.25)
- Once/twice a year	5 (15.2%)	111 (16.3%)	
- Never	24 (72.3%)	474 (69.6%)	

A significantly higher proportion of farms with redwater cases had participated in the Rural Environmental Protection Scheme (REPS) in the past. However, there was no correlation between babesiosis incidence and the presence of a specific habitat under national environmental protection on the farm. According to the farmers’ own observation, there was also no difference in the abundance of deer on farms with or without cases.

### Babesiosis diagnosis and presentation

Farmers reported that about 40% of cases occurred in both spring and summer, after which the prevalence declined to 19% in autumn and just over 3% in winter. According to veterinary practitioners, on the other hand, there was a distinct peak of 48% in summer with lower prevalence rates in spring (20%) and autumn (20%). They also reported a small number of cases in winter, with 11% of veterinarians stating that they expected to see cases at any time of the year.

The vast majority of infections were identified by case history (43%) or a previous history of babesiosis on the farm (35%). Identification by ‘treatment success’ was ticked in 18% of cases. Blood smear examination and referral to a regional veterinary laboratory were employed in only a small number of cases (3 and 1% respectively).

Farmers listed haemoglobinuria (32%) and sluggishness (25%) as the predominant clinical signs, followed by fever (16.5%) and constipation (13%). Jaundice (8%) and diarrhoea (6%) were reported least often. A very similar pattern emerged among veterinarians who distinguished haemoglobinuria (37%) and tachycardia (26%) as the most common clinical signs of bovine babesiosis, followed by fever (13%), constipation (10%) and tachypnoea (6%). Only a few veterinary practitioners listed diarrhoea (5%) or jaundice (3%) as chief presenting signs. Several veterinarians had observed changed, aggressive behaviour in affected cattle and suggested that this was an important issue for animal handlers attending cases.

According to the farmers’ survey, just under half of all cases were attended by a veterinary surgeon (46%), while veterinarians stated that, of the cases they attended, on average 26% had to be revisited and 33% required blood transfusions. Estimated mortality rates ranged between 14.3% (farmers) and 21% (veterinarians). Furthermore 2% of farmers and 23% of veterinary practitioners thought that the severity of bovine babesiosis had increased in the last decade. No change was reported by 23% of farmers and 43% of veterinarians, while the remainder either thought that severity had decreased (7% of farmers, 12% of veterinarians) or they did not know (68% of farmers, 23% of veterinarians).

### Treatment and control

All veterinary practitioners but only 20% of farmers reported that they found treatment with imidocarb effective or highly effective, although some veterinarians suggested effectiveness might have decreased over the last 15 to 20 years. Many pointed out that effectiveness of the drug was highly dependent on the timing of treatment and several voiced concerns over the toxicity of the drug. No knowledge of the efficacy of imidocarb was reported by 78% of farmers, while just over 1% thought it was not effective.

About 80% of veterinary practitioners and 4% of farmers used imidocarb prophylactically, although some said that the period of protection afforded by the drug appeared to have declined which meant that there was frequently not sufficient time for immunity to develop. Both veterinarians and farmers named application of synthetic pyrethroids (particularly flumethrin, marketed under the trade name Bayticol, Bayer Ltd) as the preferred control measure. Pour-on macrocyclic lactone (ML) preparations were also identified as tick control measures.

### Annual sales of Imizol and Bayticol

According to industry figures, annual sales of Imizol have declined from 3,355 100 ml doses in 2004 to 2,326 in 2012 (GFK-Ireland, personal communication). Over the same period sales of Bayticol have gradually increased from 3,921 (2004) to 5,437 1% 1 l solutions (2012). Unfortunately pre-2004 figures were unavailable.

## Discussion

According to the literature, *B. divergens* occurs all over Northern and Central Europe and may extend as far as Northern Africa (reviewed by Zintl and others [3]). Together with other bovine *Babesia* spp, it is thought to be of major economic importance as mortality due to acute infection, ill-thrift, abortions, loss of milk and meat production and the implementation of control measures represent a significant cost to the livestock industry [17]. Several decades ago bovine babesiosis was also considered an important disease affecting the Irish cattle industry. During the 1980’s about 1.7% of the national herd and almost 30% of farms were affected causing estimated losses of €7.6 million each year [18,19]. Within the next ten years a gradual decline in the incidence of clinical disease was noted from an estimated annual incidence of 0.82% in 1990 to 0.45% in 1994 [11]. In 2013 we carried out another survey to determine whether case rates have continued to fall. While the north west of the country was somewhat underrepresented, our results indicate that the incidence of bovine babesiosis has continued to decline (a prevalence of approx. 0.06% p.a. with about 5% of cattle farms affected were estimated, however, these figures have to be considered with caution due to the small sample size). Perhaps even more tellingly, a large percentage of farmers and a small but significant number of veterinarians confessed ignorance in relation to possible recent changes in the epidemiology, severity of disease or effectiveness of treatment. A similar decline in the prevalence of clinical redwater cases has been reported in Norway, where it was explained by decreased cattle pasturing and in Hungary, where cattle densities in endemic areas have been reduced by two-thirds while average sunlight hours per year have markedly increased [12].

Though widespread, bovine babesiosis is usually localized to specific areas where the particular environmental requirements of the vector are met. Similarly in Ireland areas in the west and northwest and the Shannon river system have traditionally been considered infection hotspots, while the southeast had the lowest prevalence ( [19], Figure 1c). This distribution was thought to be largely a reflection of the quality of grazing, as suitable tick habitat represented by inferior, marginal land, is most common in the west and northwest. Because this type of land was mainly used for beef suckling herds and store cattle, these enterprises traditionally experienced the largest number of babesiosis cases.

Our survey of veterinarians showed a similar geographical distribution. In contrast, cases reported by farmers did not display any foci of infection but were dispersed throughout the country. This may have been due to the fact that very few beef farmers participated in our survey and coverage in the northwest was lower than in any other part of the country. It is therefore possible that our annual incidence rate somewhat underestimates the actual prevalence of the disease. However, even among veterinarians the average number of cases attended each year was about 10, a reduction of over 90% from the 1980’s [19]. In relation to the type of farms most affected, previous surveys found that smaller operations had the highest infection levels. It was suggested that this was due to a lack of capital that would allow farm improvements (such as clearance of shrubs, land drainage and general improvement of rough pasture) which could reduce tick densities [19,20]. In our survey, there was no difference between the size of herds that had experienced cases of clinical babesiosis and of those that had not, indicating that the link between socioeconomics and babesiosis disease risk may no longer exist. However due to the general increase in herd size, the smallest herd size category in our survey included significantly larger herds (1–99 head) than in previous surveys (small: 20–39; medium: 44–59 and large: 60+ head).

The recently discontinued State-funded Rural Environmental Protection Scheme aimed to support environmentally sustainable farming practices including the protection of riparian zones and wild bird habitat. Specifically the scheme prescribed the maintenance of farm and field boundaries and regulated the use of herbicides, pesticides and fertilisers in and around hedgerows, lakes, ponds, rivers and streams [21]. Interestingly a higher proportion of farms with babesiosis cases had taken part in this voluntary scheme in the past. It is unclear whether participation inadvertently led to an increase in tick habitat on the farmers’ land or whether the correlation is due to confounding factors such as a greater participation in the REPS scheme by farmers with marginal land, or a better overall awareness of REPS farmers of the health status of their herd. There was no correlation with the presence of specific habitats under National or European environmental protection on the land (e.g. Natural heritage areas, Special areas of conservation or Special protected areas) some of which are likely to provide highly suitable tick habitat. While there was also no correlation with the presence of deer on the land, it is important to stress that our figures were based entirely on the farmers’ own observations and as such were dependent on factors like the distance of the land from the farmhouse and the type of enterprise, as dairy farmers tend to observe their cattle more frequently and regularly than beef farmers. It is thought that deer are the preferred host of ticks, particularly adult instars, and that their presence in an area is often positively correlated with tick densities [22]. However, while parasites that are closely related, if not identical, to *B. divergens* have been found in red deer (*Cervus elaphus*), this deer species has a restricted distribution in Ireland. The commonest deer species, fallow deer (*Dama dama*) is not thought to be a competent reservoir for *B. divergens*[23].

Previous work found that in Britain and Ireland babesiosis cases follow a distinct bimodal seasonal distribution, with a pronounced peak in May and June, a trough during midsummer, and a second, lower peak in autumn [6,24,25]. This distribution was thought to reflect the feeding activity of the tick vector, which occurs as spring and autumn-active populations [24]. Our survey indicated a different pattern with a single peak in spring/summer, absence of a summer trough and significant numbers of cases reported at any time of year including winter.

Diagnosis relies chiefly on clinical presentation with haemoglobinuria, tachycardia and sluggishness listed as the predominant signs. The fact that both farmers and veterinarians described constipation as a more important presenting sign than diarrhoea, indicates that many infections are only detected after they have progressed to the advanced stage. This is borne out by the high rate of revisits and blood transfusions. Moreover mortality rates reported in our survey were considerably higher than in the 1980’s (10 to 12.5%) [19,26]. Nevertheless, the percentage of cases attended by veterinary practitioners, estimated at just below 50%, has remained unchanged since that time [19].

Concerns in the past about resistance development against imidocarb [18] appear to be unfounded to date. It is far more likely that in the small number of cases that reported a lack of drug effectiveness, treatment was initiated too late.

The concept of imidocarb prophylaxis relies on high infection pressures and levels of transmission, to ensure development of adaptive immunity during the three to four weeks’ protection afforded by the drug. It is likely that in areas where veterinarians reported the drug did not provide protection long enough, tick densities were low and infection rates sporadic, rendering the method unreliable. Nevertheless, as many as 80% of veterinarians said they used the drug prophylactically. However, by far the most popular control measures among veterinary practitioners and farmers were pour-on applications of the acaricide flumethrin and other synthetic pyrethroids (e.g. cypermethrin, deltamethrin). Interestingly, MLs were also listed as a control option, although ticks are not included as target species in the product claims. While the anthelmintics were shown to depress reproduction of ticks *in vitro* there is no convincing evidence to date that they are effective against tick infestation in the field [11].

## Conclusions

Our survey strongly suggests that the incidence of bovine babesiosis in Ireland has continued to decline. This conclusion is supported by the reduction in the annual sales of imidocarb. Similar reductions in *B. divergens* cases have been reported from the UK [27], Hungary [12] and Norway [13]. A variety of possible reasons have been suggested including climate change and measures taken to clear land and ‘improve’ pastures leading to a significant reduction in suitable tick habitat. A trend in the reduction of pasturing cattle, changes in the frequency of cattle transport and a move away from keeping ‘store’ cattle, which are at high risk from babesiosis as they are frequently maintained on inferior land until sold for finishing, have all been identified as important factors. Often farmers have learned to avoid affected land or use it for different livestock or crops. It has also been hypothesized that the widespread and indiscriminate use of MLs may have had the fortuitous side effect of reducing tick numbers although there is no experimental evidence for their effectiveness against ticks. It must also be remembered, that there is no direct relationship between tick numbers and redwater cases [24] and that small scattered pockets of tick populations may constitute a greater disease threat as they do not support the establishment of enzootic stability.

It is likely that in each location different interacting factors are at play causing a reduction in infection pressures. Unfortunately, since we are unable to pinpoint the specific causes, we can only hope that the current trend is set to continue. This is particularly important as herds in formerly endemic areas are losing their protective immunity to disease.

## Abbreviations

ML’s: Macrocyclic lactones; REPS: Rural environmental protection scheme.

## Competing interests

The authors declare that they have no competing interests.

## Authors’ contributions

AZ designed and carried out the survey and drafted the manuscript. GMcG participated in the design of the study and performed the statistical analysis. LO’G, JF, KD participated in the design of the survey questionnaire and helped to promote participation by contacting farmers and vets. DR collated data on sales of Imizol and Bayticol. MC and JG initiated the study, participated in the design of the survey questionnaire and reviewed the manuscript. All authors read and approved the final manuscript.

## Supplementary Material

Additional file 1 Farmers' survey questionnaire. Click here for file

Additional file 2 Vet survey questionnaire. Click here for file
