# Supplementary material for: Changing incidence of bovine babesiosis in Ireland
Source: Ir Vet J. 2014 Sep 5;67(1):19. doi: 10.1186/2046-0481-67-19 (PMC4179216; doi:10.1186/2046-0481-67-19)
Supplement: Additional file 1 — Farmers' survey questionnaire. [file 2046-0481-67-19-S1.pdf]

1. Farm details (strictly for the purpose of this survey only)

Letter and first 3 digits of your herd number

2. Approximate number of animals in the various categories (May 2013):

Beef cows

Dairy cows

In-calf heifers

Calves

Other young stock

Bulls

Sheep

Other stock (please specify)

3. Has your herd size changed in the last 2 years?

- Yes, it has increased
- Yes, it has decreased
- No, the herd size has remained pretty stable

Other (please specify)

4. How many animals have been bought in in the last 12 months?

5. Has your farm or parts of your farm ever been on the Rural Environmental Protection Scheme (REPS)?

- Yes
- No
- Don't know

6. Is your farm or parts of your farm protected under specific environmental legislation (e.g. Special Area of Conservation (SAC), Special Protected Area (SPA) or Natural Heritage Area (NHA))?

- Yes
- No
- Don't know

7. Do you ever observe deer on your land or in the vicinity?

- Yes, very frequently (about weekly)
- Yes, quite frequently (about once a month)
- Yes, infrequently (only every couple of month)
- Yes, very infrequently (once or twice a year)
- Never

8. Roughly how many cases of bovine babesiosis (redwater) did your herd experience in the last 12 months?

9. At what time of year did these cases occur?

Approx. number of clinical cases in the spring

Approx. number of clinical cases in the summer

Approx. number of clinical cases in the autumn

Approx. number of clinical cases in the winter

10. What were the chief clinical signs?

	most often	sometimes	rarely	never
Urine with a red-brownish color				
High temperature				
Changes in the colour of the gums and under the eyelids from pink to abnormally pale or a yellow tinge (jaundice)				
Diarrhoea				
Constipation				
Sluggish movement				

Other (please specify)

11. How many of these cases

were attended by a veterinary practitioner?

resulted in death?

12. Do you think there has there been a change in the number of clinical babesiosis cases in the last 10 years?

- Yes, they have increased
- Yes, they have decreased
- No, they have not changed
- Don’t know

Comment

13. Do you think there has there been a change in the severity of bovine babesiosis cases in the last 10 years?

- Yes, infections tend to be more severe
- Yes, infections tend to be less severe
- No, the severity has not changed
- Don't know

Comment

14. How effective is treatment of babesiosis (redwater) with Imizol (imidocarb diproprionate)?

- Highly effective
- Effective
- Not effective
- Not at all effective
- Don't know

Comment

15. Do you use Imizol prevent redwater (prophylactically)?

- Yes
- No

16. If yes, how effective is the drug in preventing the disease?

- Highly effective
- Effective
- Not effective
- Not at all effective

17. Do you use any other drugs to prevent redwater? If yes, please specify:

18. Would you be amenable to receive an email seeking further information? If yes, please provide your name and contact details:

Name

Email or telephone number

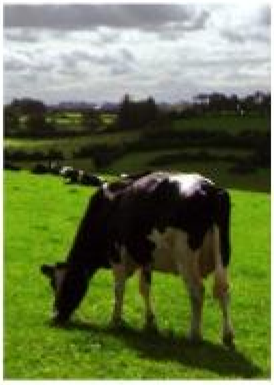

Many thanks for taking the time to complete this survey!
