# Supplementary material for: Changing incidence of bovine babesiosis in Ireland
Source: Ir Vet J. 2014 Sep 5;67(1):19. doi: 10.1186/2046-0481-67-19 (PMC4179216; doi:10.1186/2046-0481-67-19)
Supplement: Additional file 2 — Vet survey questionnaire. [file 2046-0481-67-19-S2.pdf]

1. Location of your practice:

Town

County

2. Roughly how many cases of bovine babesiosis (redwater) did you attend in the last 12 months?

3. At what time of year did they mostly occur?

- Mainly in the spring
- Mainly in the summer
- Mainly in the autumn
- Mainly in the winter
- At any time of year

Other (please specify)

4. Approx how many of these cases:

a) Needed to be revisited?

b) Required blood transfusions?

c) Resulted in mortalities?

COMMENTS

5. What were the chief presenting clinical signs?

	most often	sometimes	rarely	never
Haemoglobinuria (redwater)				
Fever				
Jaundice				
Diarrhoea				
Constipation				
Rapid breathing (tachypnea)				
Increased heart rate				
Edema of the brisket				

Other (please specify)

6. Do you think there has there been a change in the number of clinical babesiosis cases in the last 10 years?

- Yes, they have increased
- Yes, they have decreased
- No, they have not changed
- Don't know

Comment

7. Do you think there has there been a change in the severity of bovine babesiosis cases in the last 10 years?

- Yes, infections tend to be more severe
- Yes, infections tend to be less severe
- No, the severity has not changed
- Don't know

Comment

8. How are most cases of bovine babesiosis diagnosed? Please rank according to your experience

	most often	sometimes	rarely	never
Case history				
Previous history of babesiosis on the farm				
Treatment success				
Blood smear (in-house)				
Referral to a regional laboratory				

Other (please specify)

9. How effective is treatment of bovine babesiosis with imidocarb diproprionate (Imizol)?

- Highly effective
- Effective
- Not effective
- Not at all effective
- Don't know

Comment

10. Do you use Imizol prophylactically?

- Yes
- No

11. If yes, how effective is the drug as a prophylactic agent?

- ○ Highly effective
- ○ Effective
- ○ Not effective
- ○ Not at all effective

12. Do you use any other drugs as prophylactic agents against bovine babesiosis? If yes, please specify:

13. Would you be amenable to receive an email seeking further information? If yes, please provide your name and contact details:

Name

Email/Telephone

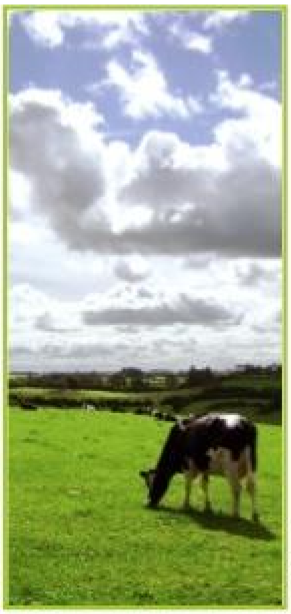

Many thanks for taking the time to complete this survey!
